# Application of CRISPR-Cas9 gene editing technology in basic research, diagnosis and treatment of colon cancer

**DOI:** 10.3389/fendo.2023.1148412

**Published:** 2023-03-20

**Authors:** Hui Meng, Manman Nan, Yizhen Li, Yi Ding, Yuhui Yin, Mingzhi Zhang

**Affiliations:** ^1^ Department of Pathology, First Affiliated Hospital of Zhengzhou University, Zhengzhou, Henan, China; ^2^ Department of Oncology, First Affiliated Hospital of Zhengzhou University, Zhengzhou, Henan, China

**Keywords:** CRISPR tools, genome editing, colorectal carcinoma, Genetic tool, basic research

## Abstract

Colon cancer is the fourth leading cause of cancer death worldwide, and its progression is accompanied by a complex array of genetic variations. CRISPR/Cas9 can identify new drug-resistant or sensitive mutations in colon cancer, and can use gene editing technology to develop new therapeutic targets and provide personalized treatments, thereby significantly improving the treatment of colon cancer patients. CRISPR/Cas9 systems are driving advances in biotechnology. RNA-directed Cas enzymes have accelerated the pace of basic research and led to clinical breakthroughs. This article reviews the rapid development of CRISPR/Cas in colon cancer, from gene editing to transcription regulation, gene knockout, genome-wide CRISPR tools, therapeutic targets, stem cell genomics, immunotherapy, metabolism-related genes and inflammatory bowel disease. In addition, the limitations and future development of CRISPR/Cas9 in colon cancer studies are reviewed. In conclusion, this article reviews the application of CRISPR-Cas9 gene editing technology in basic research, diagnosis and treatment of colon cancer.

## 1 Introduction

In 2020 the Nobel Prize for chemistry was awarded to Emmanuelle Charpentier and Jennifer Doudna (1), for their development of palindromic/CRISPR-associated nucleic acid 9 (CRISPR/Cas9) gene editing technology, providing new tools for accurate gene editing (2, 3). Virtually any genomic site can be targeted using a single complex nuclease protein with a short RNA as a site-specific endonuclease.

There are three types of genome editing, such as zinc-finger nucleases, transcriptional activator-like effect nucleases (TALENs), and CRISPR/Cas9. CRISPR/Cas9 is simple to use and is the most dominant and popular technique in genetic engineering (4). Gene editing holds great promise for cancer treatment by tweaking gene expression and correcting mutations, This could lead to further breakthroughs in the field of precision oncology.

Colon cancer is generally thought to be clonal, involving sequentially progressive genetic events involving loss of tumor suppressors (e.g. APC and p53) and increased function of oncogenes (e.g. K ras, SMAD4, etc.) (5).

Colorectal cancer can be divided into four categories. Subtype 1 is associated with microsatellite instability (MSI), subtype 2 is more prone to hypermethylation and favorable prognosis, as well as Wnt signaling pathway activation and TP53 mutation, and K ras mutation in type 3 tumors, Subtype 4 shows activation of TGFβ pathway in response to matrix infiltration (6).

Carcinoma *in situ* (Intraepithelial neoplasia, CIN) is present in about 85% of sporadic colorectal cancers. Carcinogenesis is caused by defects in telomere stability, chromosome separation, DNA damage response, chromosome rearrangement and loss of heterozygosity (LOH) of tumor suppressor genes, mainly involving APC, TP53, DCC and members of the SMAD family (SMAD2 and SMAD4) (7).

MSI occurs in about 15-20% of sporadic CRCs, which arises from epigenetic silencing or germline mutations in one of the MMR genes and is characterized by a disorder of the DNA mismatch repair (MMR) system, resulting in a high frequency of nucleotide insertions/deletions in microsatellite DNA repeats (8).

Cytosine-phosphate-guanine (CpG) island methylator phenotype (CIMP), characterized by epigenetic changes such as promoter methylation and gene silencing, was found in 17% of CRCs (9).

Epigenetic changes in colon cancer also involve RNA alterations, which are characterized by dysmethylation (due to altered expression of specific enzymes) and are associated with overactivation of the MAPK/ERK and Wnt/β-catenin pathways (10).

The advent of new high-throughput “omics” technologies has added complexity to the biological characterization of CRC. In addition to understanding the evolution of their genetic heterogeneity, the use of transcriptomics has also identified clearly distinct subtypes of CRC, termed shared CRC molecular subtypes (CMS). Tumor, stromal and immunologic components as well as classic histopathological classification were included (11, 12).

**Figure 1:**
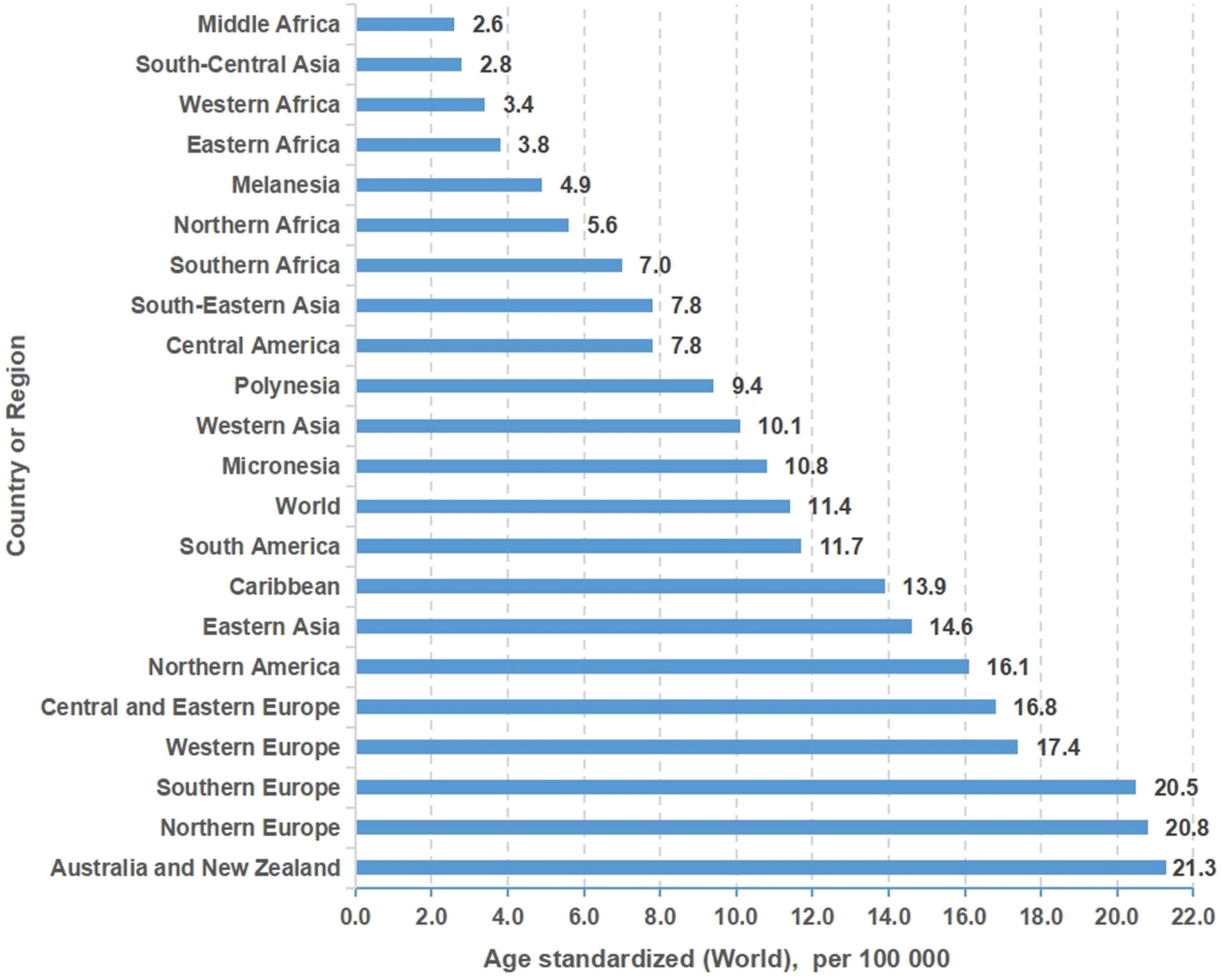
Incidence of colon cancer in different regions or countries around the world.

Colon cancer is generally thought to be clonal, involving sequential progressive genetic events. Colorectal cancer is the fourth leading cause of cancer death globally and is highly resistant to both classical chemotherapy agents and new targeted agents (**Figure 1**). There is an urgent clinical need to develop new high-throughput approaches, including genome-wide, CRISPR/Cas9, and chemical approaches, to identify new targets and develop relatively specific inhibitors that sensitize resistant tumors or target mutations with specific carcinogenicity.

Gene editing technology includes gene knockout, gene directed mutation, gene fusion, multi-target knockout, and multiple editing technology. In this paper, gene knockout technology, which occupies a large proportion in gene editing technology, is described respectively. Meanwhile, the deep application prospect and importance of CRISPR gene knockout technology are also considered.

Use CRISPR/Cas9 gene knockout, CRISPR/Cas9 gene editing, and CRISPR/Cas9 libraries to discover therapeutic targets or novel approaches to new drugs, and identify new actionable targets (**Table 1**), some of which have been tested in clinical trials, may lead to a significant improvement in the treatment of colon cancer patients shortly, especially in the context of personalized approaches.

**Table 1:** CRISPR/Cas9 techniques and colon Cancer.

Type	Gene modified	References
knockout	PUM1 and DDX5; WHSC1; AP-2α; CYSLTR1; FGFR1 and OXTR; SASH1; Par3L; caspase-3; NR1H4; uPAR; HNRNPA2B1; GPVI; NSD2; p38γ; CATSPER1; NMRAL2P; HABP4; ZAP and APC; PRDM1; LDHA and LDHB; NAT1; SDHB; VDR; PPIP5KS; NOXO1; PLAUR; PPIP5K; NHLRC2; BrafV600E; LIF; CXCR-4; SGPL1; DACH1; CD133; APC; RNF43; H3K27Ac; GUCY2C; METTL3; CD44 and TLR4; CCAT2; CBS; TGM2; STAT3; mindin; GSTO1 and αIP3/A20; COMP; TRIM11; MBTPS1; RhoB; CD44v6 and YB-1; Crb3; PP2A; Bid; USP29; CD133; Wnt-1; NHE8; P-cadherin; IL-6; SPHK1; IDO-1; ILK; AH1; SIAE; CD133; Nrp2; Tks4; p53 R273H; ALDOB; SNPs	(13–78)
gene editing	Rspo2 and Rspo3; APC, Smad4, Tp53 and KRAS; rs11064124; KRAS, MEK1, PIK3CA and MTOR; TP53; APC; KRAS and TP53; APC; CXCR2 and IL-2; MLH1; β-catenin; IL12 and CXCL11; MUC5AC; VEGF-A; HuR; ERO1α; PC; αTAT1; MARCH2; Klotho; FAPP2; CLCA1; rs4779584 and rs1406389; rs34149860; rs6854845; p53	(77–101)
CRISPR library	HMGCR, FDPS and GGPS1; GRB7; ZEB2; β-catenin; KRAS; CCT6A, RHOQ, RRP12, UTP18, DDOST, YRDC, ACTG1, RFT1 and NLE1; CRS; ERN1; LGALS2; NFKBIZ, ZC3H12A and PIGR;/; TRAF5	(102–113)

This article describes how CRISPR/Cas gene editing technology opens up new avenues for the research, diagnosis, and treatment basis of colon cancer. The CRISPR/Cas9 genome editing tool has been used effectively in colon cancer biology, as outlined below.

## 2 CRISPR/Cas9 system

### 2.1 Classical CRISPR/Cas9 system

**Figure 2:**
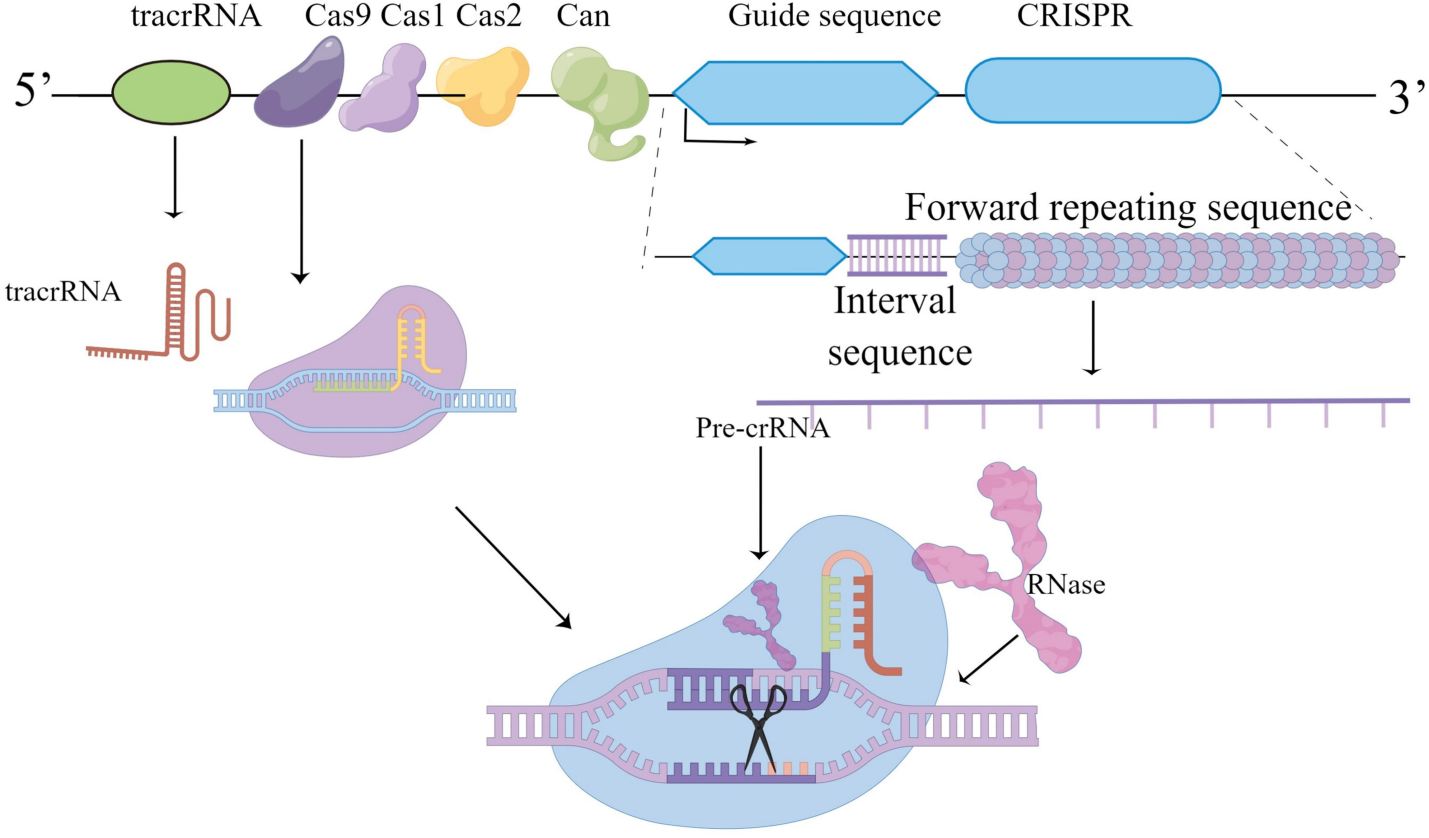
Graphical representation of CRISPR/cas9 about its working and applications.

CRISPR/Cas9 components found in eubacteria and arachnoid membranes disrupt invading phage DNA/RNA. CRISPR/Cas9 acts as a molecular scissors, and the orthologous gene accompanying the CRISPR locus is the Cas gene, which encodes an endonuclease. The CRISPR/Cas system is similar to mammalian RNAi, in which CRISPR loci are transcribed into mature CRISPR Rnas (crRNAs). The maturation of crRNA is mediated by trans-activating crRNA (tracrRNA), endonuclease Cas9 and RNase III leading to molecular cleavage. The bacterial CRISPR/Cas9 system as a powerful genetic tool, coupled with its ease of use, makes it a very popular technology for a wide range of applications in life sciences (114) (**Figure 2**).

About 93 Cas genes are known, the most efficient of which is Cas9 endonuclease, which cleaves DNA. Other Cas proteins such as Cas13 can cleave RNA (115).

The acronym CRISPR refers to the clustered regularly spaced short palindromic repeat of 24-40nt, first identified as a unique ordered repeat in E. coli and Haloferax Mediterraneum (116, 117).So the discovery that CRISPR/Cas9 is like molecular scissors is being used effectively as a tool for genome editing. The CRISPR/Cas9 component originally evolved as a powerful innate defense mechanism against viral infection, helping to destroy invading phage DNA/RNA. Similar sequences were later found in other bacteria and arachnids. The homologous gene accompanying the CRISPR locus is the Cas gene encoding endonuclease (CRISPR-related gene). Bacteria carrying similar phage spacers can resist infection by phages carrying the same sequence, leading to the hypothesis that the CRISPR/Cas system is similar to mammalian RNAi. CRISPR/Cas is a technique that allows researchers to cut DNA at a specific point and then edit the genome.

### 2.2 The CRISPR/Cas9 system and the development

In 1987, Japanese scientists found some unknown in the e. coli genome tandem repeat sequences, but the meaning of these sequences was unknown at that time (116). The role of CRISPR loci in adaptive immunity was discovered, and it was speculated that protospacer adjacent motif (PAM) might direct Cas9 type II nucleases to cut DNA (118–120). Trans-coding crRNA (tracrRNA) was involved in the processing and maturation of pre-crRNA (121), and their research revealed new pathways maturation of crRNA. Mature crRNA pairs with tracrRNA to form a special double-stranded RNA structure through base complementarization, which guides the Cas9 protein to cause double-stranded breaks in the target DNA (122). The application of the type II Cas system to DNA cutting in mammalian cells paves the way for the use of the CRISPR/Cas9 system in genome modification (123). Several initiatives have ameliorated the problem of unexpected interruptions in the CRISPR/Cas9 gene-editing system (124–126). CRISPR/Cas9 variation in the process of change, some drive research developed SpCas9 variations, almost entirely freed from the constraints of PAM sequence, expanded the use of CRISPR/Cas9 systems in genome editing (5).

Cas12a offers a new approach to genome editing with a unique cutting mechanism that enhances and extends the CRISPR Tool kit (127). Unlike Cas9, the enzyme Cas12a recognizes the T-rich procapsule adjacent motif (PAM). Cpf1-associated CRISPR arrays are processed into mature crRNA without the need for additional trans-activated crRNA (tracrRNA) (121). Cpf1 introduces staggered DNA double-strand breaks with 4 or 5-nt-5 ‘suspensions. Cas12a is an RNA-directed DNA-targeting enzyme capable of inducing genetic changes in cells at double-stranded DNA (dsDNA) cleavage sites. Cas12a can be used as a genome editing tool powerful enough to fully degrade both linear and circular ssDNA molecules. Cas12a and related type V CRISPR interfering proteins release non-specific single-stranded DNase activity after guiding RNA-dependent DNA binding, which can be used for rapid and specific nucleic acid detection. Cas12a/Cas12b is suitable for *in vivo* delivery of adeno-associated virus (AAV) -mediated gene therapy, significantly expanding the targeting range of the genome for genome editing, gene activation, and generation of mutant animal models, further expanding the scope of CRISPR/Cas9 applications (128).

The CRISPR/Cas13 system allows cut RNA bands to form visual cues and display them visually (115). It is cheaper, faster and has improved the efficiency of testing for viruses. Notably, the researchers found that the CRISPR/Cas13 gene-editing system delivered by the adeno-associated virus AAV can edit and remove the novel coronavirus (129, 130). In conclusion, Cas13 may be a potential clinical diagnostic and therapeutic tool for viral infections.

## 3 Application of the CRISPR/Cas system in colon cancer research: Knockout technique

**Figure 3:**
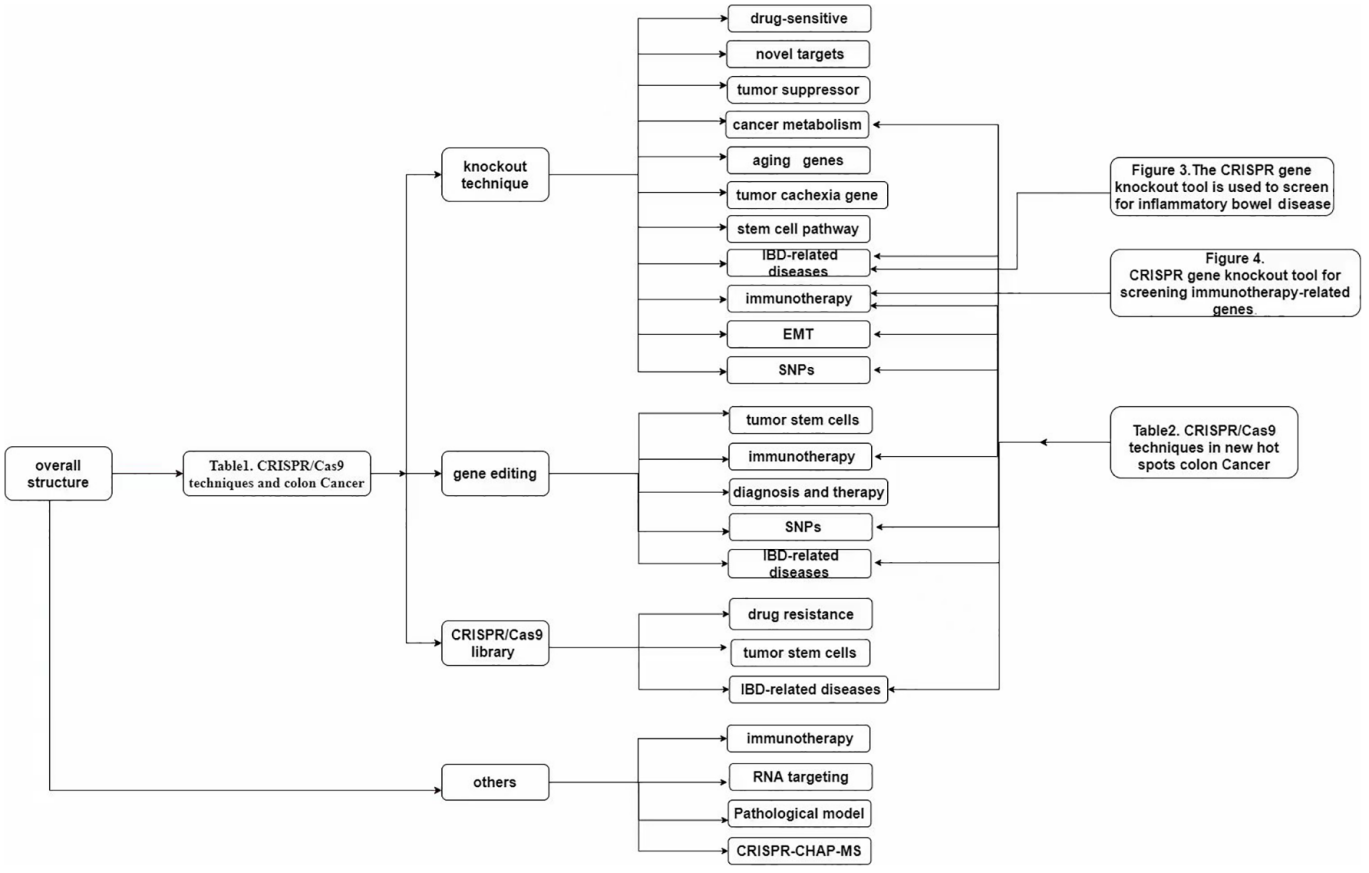
Overall structure.

The use of CRISPR gene knockout editing technology will bring feasible strategies for clinical targeting of tumor genes, including targeting tumor suppressor genes and tumor oncogenes, identifying new cancer therapy target genes related to CSC, and understanding abnormal glucose metabolism, lipid metabolism, amino acid metabolism, mitochondrial biogenesis and energy metabolism abnormalities including metabolic reprogramming pathway in cancer cells (**Figure 3**) (131, 132).

### 3.1 Study of colon cancer drug-sensitive target genes using CRISPR/Cas knockout technique

Liu et al. demonstrated the role of PUM1 in the mechanism of colon cancer resistance by knockout PUM1 and dead-box helicase 5(DDX5) in trastuzumab-resistant SW480R and Caco-2R cells and found that the proliferation capacity of both types of cells was reduced. It was also found that PUM1 can positively regulate (DDX5) and increase cell viability, thus proving that the downregulation of PUM1 and DDX5 can reduce tumor cell viability and is a therapeutic target for the increased sensitivity of colon cancer to trastuzumab (13).

Liu et al. studied the interaction between HMGA2-WHSC1 in the regulation of cancer cell growth and found that WHSC1 is a transcriptional target of the oncogene HMGA2, which can promote cancer cell proliferation and metastasis.CRISPR knockout of WHSC1 in colon cancer cells inhibits colon cancer cell proliferation, increases drug sensitivity, and decreases metastatic ability (14).

Activated protein 2α (AP-2α) encodes a tumor suppressor by TFAP2A, which regulates colon cancer transcription. Becket al. used CRISPR/Cas9 and short hairpin RNA to eliminate TFAP2A expression in HCT116 and a group of colon cancer cell lines. Tumor cells become resistant to the PI3K inhibitor buparlisib BKM120 after AP-2α knockout, suggesting that up-regulation of AP-2α may increase sensitivity to BuparlisiB/BKM120 (15).

Satapathy et al. studied the drug resistance of cysteamine leukotriene receptor 1 in 5-FU. CYSLTR1 level increased in 5-FU resistant cells, both CYSLTR1 genes were knocked out and Montelukast reduced the signal transduction of LTD4/CYSLTR1, and the resistance to 5-FU was weakened (16).

Tung et al. found that tumor cells develop a tolerance to oxaliplatin due to chromatin alterations, fibroblast growth factor receptor 1(FGFR1) and oxytocin receptor (OXTR) are genes associated with increased chromatin accessibility, CRISPR/Cas9 silencing fibroblast growth factor receptor 1 (FGFR1) and oxytocin receptor (OXTR) helps overcome oxaliplatin resistance. Similarly, treatment with oxaliplatin in combination with an FGFR1 inhibitor (PD166866) or an antagonist of OXTR (L-368,899) suppressed chemoresistant organoids. This provides a feasible method for the precision treatment of colon cancer (17).

The study of Franke et al. showed that the low expression of SASH1 was related to distant metastasis of tumors. SASH1 expression is low and CRKL expression is high in colon cancer cells. SASH1 knockout can induce EMT. As a result, the sensitivity of the tumor to chemotherapy drugs and the survival of patients is reduced (18).

Zhou et al. found through *in vitro*, *in vivo*, subcutaneous, and intravenous injection experiments that compared with the control group, HCT116 cells with caspase-3 gene knockout showed increased sensitivity to chemotherapy and radiotherapy, and weakened tumor invasiveness and metastasis ability, suggesting that caspase-3 can be used as a therapeutic target for colon cancer (19).

Li et al. suggest that Par3L is a potential therapeutic target for colon cancer treatment. Colon cancer patients with high Par3L expression had lower survival rates. Using the CRISPR/Cas9 gene knockout technique, the apoptosis of colon cancer cells with deletion of Par3L expression was increased. In addition, the deletion of Par3L leads to abnormal activation of the Lkb1/AMPK signaling cascade, leading to increased sensitivity of colon cancer cells to chemotherapy and radiotherapy (133)..

### 3.2 CRISPR/Cas9 knockout technique: Identification of novel colon cancer targets

Nuclear receptor subfamily 1 group H member 4 (NR1H4) plays an important role in the proliferation and survival of colon cancer. It was found that NR1H4 KO enhanced the anticancer effects of adriamycin and cisplatin. MYC is an important mediator of NR1H4 KO-induced signaling pathway changes, and inhibition of NR1H4 activity in colon cancer cells can be used as a choice for targeted therapy (21).

Biagioni et al. investigated the effect of uPAR gene knockout on the proliferation of colon cancer cells. Knockout of the uPAR gene by CRISPR gene knockout technology can lead to tumor cell apoptosis, increase the sensitivity of tumor cells to chemotherapy drugs, and down-regulate EGFR expression, suggesting that uPAR can be used as a therapeutic target for colon cancer (134).

HNRNPA2B1 activation can promote tumor cell growth, and this RNA-binding protein can activate the ERK/MAPK pathway, thereby regulating tumor cell apoptosis. Tang et al. verified the mechanism of HNRNPA2B1 in tumor cells through immunohistochemistry and western blotting. and the growth of SW480 cells was inhibited after gene knockout (135).

Glycoprotein VI (GPVI) triggers platelet activation and is a safe target for antithrombotic action. Recent studies have revealed the metastasis mechanism of glycoprotein VI in breast and colon cancer. Mammadova-Bach et al. demonstrated reduced tumor metastasis in mice with platelet GPVI gene deficiency. Gene knockout method confirmed that platelet GPVI interacts with Galectin-3 on tumors to promote tumor metastasis. We also found that AQ1 F (ab ‘) 2-mediated GPVI inhibition inhibited tumor metastasis by inhibiting tumor-platelet interaction (24)..

The nuclear receptor binding domain protein 2 (NSD2) is a key histone methyltransferase, and its expression in colorectal cancer is related to the proliferation and migration of colorectal cancer. Zhao et al. studied the mechanism and function of NSD2 in colorectal cancer and found that NSD2 mRNA was elevated in colon cancer. After the CRISPR/Cas9 knockout of NSD2, the proliferation and development of colorectal cancer were inhibited, suggesting that NSD2 could be a potential target for the treatment of colon cancer (136).

p38γ (P38γ) is a new cell cycle-dependent kinase (CDK) -like kinase, which plays a certain role in tumor growth and migration. Su et al. studied the growth of tumor cells after knockout of p38γ, and found that cell growth was blocked and p38γ was highly expressed in colon cancer, suggesting that p38γ could promote the growth of colon cancer as a new therapeutic target for colon cancer (20).

CATSPER1 is closely related to the poor prognosis of colon cancer and is highly expressed in colon cancer. The mechanism of CATSPER1 may activate the PI3K/Akt signaling pathway and promote the proliferation of colon cancer cells. After the knockout of the CATSPER1 gene by Huang et al., the growth of colon cancer is inhibited. This suggests that CATSPER1 can be used as a therapeutic target for colon cancer (137).

Johnson et al. found that the knockdown of NMRAL2P *via* CRISPR/Cas9 genome editing can protect the therapeutic effect of SFN on colon cancer. NMRAL2P is a direct transcriptional target of NRF2 and a functional pseudogene regulated by NRF2 (22).

### 3.3 CRISPR/Cas9 knockout technique: Identification of tumor suppressor genes

Melo-Hanchuk et al. found that HABP4 (HABP4), a 57kDa regulatory protein, is involved in tumor transcription regulation and proliferation regulation of colon cancer. After gene knockout, tumor volume increased, indicating that HABP4 is expected to become a new tumor suppressant protein (23).

Zinc finger ZAP antiviral protein expression level is associated with the prognosis of colon cancer, ZAP ectopic expression inhibits tumor growth, proliferation, and invasion. Cai et al. used CRISPR engineering to knock out ZAP and APC and found that the deletion of both synergies promotes tumor growth (138).

Liu et al. found that PRDM1β is a p53 response gene in colon cancer cells. By knockout technique, the α and β transcriptional isoforms of PRDM1 inhibit myc response genes and stem cell-related genes, and overexpression of the PRDM1 gene could inhibit the growth of colon cancer. p53 may play a tumor suppressor role through PRDM1-dependent stem cell gene silencing (25).

### 3.4 Study of colon cancer metabolism-related genes using CRISPR/Cas9 knockout technique

Ždralević et al. showed that the Warburg effect is essential for the growth of aggressive tumors and that double knockdown of LDHA and LDHB genes can inhibit the glycolytic pathway, thereby inhibiting the growth of aggressive tumors. Cancer cells consume more glucose than normal cells, and this phenomenon of increased cancer cell glycolysis is known as the Warburg effect. Double knockout of LDHA and LDHB completely inhibited lactate secretion and cell growth at room temperature, while tumor cell growth was completely inhibited *in vitro* under hypoxic conditions (26).

NAT1 knockout abolished the expression of gain-of-function p53 protein in HT-29 cells. During glucose starvation, tumor growth was inhibited and cell apoptosis was increased after NAT1 knockout. HT-29 can regulate the growth of tumor cells by stabilizing the p53 protein (27).

SDHB gene mutation is associated with poor prognosis of tumors. After SDHB gene knockout, oxygen consumption decreased and glycolysis pathway was enhanced. The proliferation of SDHB knockout cells was inhibited by glycolysis inhibitors (28).

Irving et al. found that vitamin D did not affect the development of colon cancer. Through the gene knockout vitamin D receptor (VDR), it was found that the loss of VDR expression did not promote the occurrence of the tumor, and did not affect the proliferation and progression of the tumor (29).

Inositol 5-INSP7 (inositol pentaphosphate diphosphate) and 1, 5-Insp8 (inositol tetraphosphate diphosphate) are high-energy cell signals that are interconverted by inositol pentaphosphate kinase (PPIP5KS). Gu et al. found that 5-INSP7 can regulate the level of p53, and 1,5-INSP8 regulates ATP levels. CRISPR knockout of pPIP5Ks in colon cancer cell lines slowed cell proliferation and increased the cell cycle (30).

Joo et al. found that NoxO1 was highly expressed in colon cancer tissues. After the knockout of this gene, the growth of tumor tissues was inhibited, and NOX1 activity as well as ROS production were regulated by GRB2/CBI-mediated NoxO1 proteolysis. The activity of NADPH oxidase (NOx) is regulated by NoxO1. GRB2 interacts with NOX1, resulting in decreased ROS production, and overexpression of GRB2 leads to decreased NOX1 activity (31).

Urokinase plasminogen activator (UPA) receptor (uPAR) can promote tumor growth. Biagioni et al. used CRISPR/Cas9 gene to knock out the uPAR-encoding gene PLAUR in colon cancer cells, resulting in immature mitochondrial development, decreased glycolysis, and increased lactate secretion (32).

The metabolism of inositol diphosphate pentaphokinase (PPIP5KS) plays an important role in proliferation. Gu et al. showed that the use of CRISPR technology to knock out PPIP5K inhibited the proliferation of colon cancer cells in the simulated tumor cell microenvironment under the condition of glucose-restricted culture (139).

Nishi et al. found that in the mechanism of reactive oxygen species (ROS) -induced apoptosis, Caspase-8 can cleave NHLRC2, leading to the decrease of NHLRC2 level, and ROS overproduction can cause the caspase-8-mediated decrease of NHLRC2 protein level. Knockdown of Caspase-8 resulted in increased NHLRC2 levels and inhibited cell apoptosis (140).

### 3.5 CRISPR/Cas9 knockout technique: Identifying aging genes and tumor cachexia gene

Promoter hypermethylation mimics the human aging-like phenotype. Tao et al. found that CRISPR-mediated simultaneous inactivation of silencing genes activates the Wnt pathway. These changes make aging organs more sensitive to Braf V600E transformation than younger organs. This links aging-like epigenetic abnormalities to oncogenes that drive colon tumorigenesis (144).

Tumor cachexia is associated with a poor prognosis. Kandarian found that leukemia suppressor factor (LIF) is the main initiating factor of colon cancer cachexia. The effect of LIF expression level on tumor cachexia was detected by knockout of LIF, and it was found that weight loss, muscle loss, fat loss, and splenomegaly were improved in knockout mice (33).

### 3.6 Study of colon cancer stem cell pathway using CRISPR/Cas9 knockout technique

Zheng et al. discovered the mechanism of action of C-X-C chemokine receptor type 4 (CXCR-4) in tumor metastasis. After the knockout of (the CXCR-4) gene using gene knockout technology, the adhesion ability of tumor cells was decreased, but the proliferation ability was not affected. This effect is achieved through the regulation of AKT and insulin-like growth factor receptor type 1 (IGF1R) signaling pathways (34).

Schwiebs et al. used the CRISPR/Cas9 system to find that SGPL1 gene knockdown in the colon stem cell niche may contribute to cell cycle arrest and play a role in colon cancer progression. SGPL1 regulates cell cycle by regulating Ki-67 and FOXO3 in stem cells (35).

Hu et al. studied the mechanism of DACH1 in intestinal stem cells and colorectal tumors, and the high expression of DACH1 in colorectal cancer suggested a poor prognosis. Overexpression of DACH1 inhibits cell growth by inhibiting BMP signals. After DACH1 gene knockout, the growth inhibition is relieved. Therefore, DACH1 can be used as a therapeutic target for colon cancer (36).

Gisina et al. found that the expression level of CEACAM5 in colon cancer stem cells was associated with positive CD133. CD133 is a molecular marker of colon cancer stem cells, and the percentage of CEACAM5-positive cells varied significantly after the CRISPR/Cas9 system was used to knock out CD133 (37).

Michels et al. investigated the induction of colon cancer formation by Wnt activation in the presence of CRISPR/Cas9 knockout of APC. Oncogenic and physiological Wnt-induced transcription are distinct. In consensus molecular subtype 2 (CMS2) tumors, oncogenic Wnt signaling is associated with a good prognosis, and receptor-mediated signaling is associated with CMS4 tumors and a poor prognosis. This study helps to better understand the Wnt response in colon cancer (38).

Eto et al. investigated the effect of RNF43 deletion mutations on colon cancer progression and showed that knockdown of RNF43 using the CRISPR/Cas9 system activated Wnt signaling, accelerated tumor growth, and increased recurrence rates in patients with colon cancer (39).

Tak et al. investigated the effect of SNPs associated with CRC risk on gene expression. H3K27Ac peaks carry SNPs associated with an increased risk of CRC. CRISPR/Cas9 nuclease was used to knock down H3K27Ac in the E7 region of colon cancer cells, and the expression of many adjacent genes decreased massively. However, in colon cancer cells without an H3K27Ac peak in the E7 region, the deletion of the E7 region also reduced the expression of related genes, suggesting that the effect of enhancer deletion on transcriptome expression cannot be predicted by the size or presence of H3K27Ac peak (40).

The intestinal epithelial receptor GUCY2C regulates the tumor inhibition axis. Rappaport et al. Used CRISPR/Cas9 knockout and genome editing techniques and identified functional gene enhancers mediating GUCY2C ligand loss and investigated the mechanism of β-catenin/TCF signal transduction on transcriptional silencing of GUCY2C ligand. GUCY2C is the most sensitive target of β-catenin/TCF signaling, reflecting transcriptional repression (41).

Xu et al. showed that after METTL3 gene knockout in SW480 cells, the expression level of SOCS2 in tumor cells was increased, thereby reducing the tumorigenicity of colon cells. This prompt METTL3 tumorigenicity of colon cancer cells is a key factor (42).

Hyaluronic acid (HA) can promote the growth of colon cancer cells, and its high level is associated with poor prognosis. CD44 and TLR4 are related receptors of HA. Makkar found that the combination of HA with CD44 and TLR4 can promote the development of colon cancer, and PEP1 can block the combination of HA with receptors, and inhibit the growth of colon cancer. CT26 HA is a kind of expression of colon cancer cells, CD44 and TLR4 CRISPR knockout CT26 colorectal cancer cell was relatively slow growth, this suggests the HA combination of CD44 and TLR4 promote CT26 homologous grafts of tumor growth (43).

CCAT2 (colon cancer-associated transcription-2) is a long non-coding RNA (lncRNA) that positively regulates tumor growth, while miR-145 is a miRNA that negatively regulates gene expression. Yu et al. studied the mechanism of interaction between the two. CCAT2 is located in the nucleus, and the maturation of miR-145 begins in the nucleus. CCAT2 inhibits the movement of pre-Mir-145 from the nucleus to the cytoplasm, thereby blocking the maturation of miR-145 and promoting tumor proliferation. After the knockout of CCAT2 using CRISPR technology, the number of mature miR-145 increased, and the proliferation of colon cancer stem cells was blocked (44).

Guo et al. studied the effect of targeting the CBS-H2S axis on colon cancer liver metastasis and used the CRISPR/Cas9 system to knock out CBS. The results showed that VEGF was down-regulated, blood vessel growth was blocked, and colon cancer liver metastasis was inhibited, suggesting that CBS could be used as a therapeutic target for colon cancer liver metastasis (45).

Malkomes et al. demonstrated that Transglutaminase 2 (TGM2) has transamidation and GTPase activity. The increased expression of TGM2 in colon cancer can increase the activity of colon cancer cells, and the proliferation ability of tumors is weakened after gene knockdown of TGM2 (46).

Xiong et al. studied the effect of a short deletion in the DNA binding domain of STAT3 (STAT3DEL) on the occurrence of colon cancer. Using gene knockout technology to knock out a short sequence in the DNA binding domain of STAT3, cell proliferation and migration ability decreased. This suggests that the deletion of short sequences in the STAT3 DNA-binding domain inhibits transcriptional activation and alters the recognized DNA motif, thereby affecting colon cancer development (47).

Cheng et al. found that the deletion of mindin could promote the proliferation of cancer cells through ERK and c-fos pathways, and the establishment of mindin knockout mice through the CRISPR/Cas9 system accelerated the tumor growth of mice, while the overexpression of the mindin gene inhibited the tumor growth of mice. This suggests that mindin expression levels can regulate tumor growth and provide a target for the treatment of colon cancer (48).

GSTO1 is related to autophagy, which plays a key role in the development of drug resistance in cells. The interaction between GSTO1 and TNFαIP3/A20 promotes the generation of drug resistance. Studies have found that double gene knockout of GSTO1 and TNFαIP3/A20 using the CRISPR/Cas9 system is more sensitive to chemotherapy drugs than single gene knockout, and the apoptosis of cancer cells increases, which reveals that GSTO1 can enhance the drug resistance of cancer cells (49).

Liu et al. detected the expression level of COMP in colon cancer cells and found that COMP played a role in promoting the growth of colon cancer cells. COMP was highly expressed in colon cancer tissues, and the level of COMP decreased after colon cancer surgery. By knocking out COMP with CRISPR technology, the proliferation ability of colon cancer cells was weakened. The ability to resist chemotherapy drugs is weakened, and the cancer cells with high expression of COMP have stronger proliferation ability, resulting in poor prognosis of patients, which suggests that COMP can be used as a prognostic indicator for colon cancer and provide a new therapeutic target for the treatment of colon cancer (50).

High expression of TRIM11 (Tripartite Motif-Inplasmid protein 11), an E3 ubiquitin ligase, in colon cancer tissues led to short survival. Yin et al. used CRISPR technology to knock out the TRIM11 gene, and the proliferation of cancer cells was inhibited, resulting in cell apoptosis. Overexpression of TRIM11 inhibits cell apoptosis, and miR-24-3P regulates the expression of TRIM11. Downregulation of miR-24-3P leads to up-regulation of TRIM11, and TRIM11 can be a therapeutic target for colon cancer (51, 52).

Hartal-Benishay et al. investigated the regulatory role of MBTPS in the proliferation of colorectal cancer (CRC). Sterol regulatory element binding proteins (SREBPs) regulate cholesterol and fatty acid synthesis, thereby regulating cancer cell proliferation. The expression level of MBTPS1 mRNA in the tumor tissues of CRC patients is positively correlated with SREBPs. After the knockdown of MBTPS1, tumor growth is inhibited. Based on the knockdown of MBTPS1, the inhibition of the type 1 interferon pathway completely inhibits tumor proliferation, which suggests that MBTPS1 can be used as a therapeutic target for the treatment of colon cancer (52).

Rho protein is associated with radiation resistance in colon cancer cells. After knocking down RhoB by CRISPR/Cas9 system, Liu et al. inhibited the phosphorylation level of Akt, downregulated FoxM1, increased the sensitivity to radiotherapy, decreased the invasion ability of cells, and increased apoptosis. High expression of RhoB can increase the resistance to radiotherapy, the survival time of patients is short and the prognosis is poor (53).

Tumor-initiating cells (CICs) are associated with tumor formation and drug resistance formation. The chemotherapeutic drug FOLFOX can induce the overexpression of CD44v6, MDR1, and YB-1, the up-regulation of CD44v6 can increase the expression of YB-1, and the deletion of CD44v6 and YB-1 in the CRISPR/Cas9 system inhibits the growth of CICS in Folfox-resistant cells. CD44v6 is required for the reversal of differentiated tumor cells into tumor-initiating cells (54).

The epithelial cell polarity regulator CRUMPS3 (Crb3) is related to tumor suppression. Crb3 interacts with FGFR1, leading to its functional activation and promoting the metastasis and invasion of colon cancer (55).

Dai et al. investigated the regulatory effect of PP2A on the proliferation of colon cancer cells and found that LB-100 could inhibit the activity of PP2A, thereby inhibiting the survival and proliferation of colon cancer cells. Apoptosis was induced by the activation of AMPK by knocking out PP2A using CRISPR technology. Further, LB-100 activated apoptosis and induced G1-S cell cycle arrest in CRC cells (56).

Huang et al. investigated the effect of Bid deletion on apoptosis. Caspase8 is activated after Bid is cleaved, and Bid is generated in response to TRAIL. Bid knockout of colon cancer cells constructed by gene editing can block apoptosis (57)..

Ubiquitin-specific protease 29 (USP29) is a deubiquitination enzyme that regulates the occurrence and development of cancer. Chandrasekaran et al. studied its mechanism of action in colon cancer. Using CRISPR/Cas9 technology to knock out USP29, the cell cycle was prolonged, cell apoptosis was increased, and cell proliferation was inhibited. The high expression of USP29 can promote the occurrence and development of colon cancer and increase its malignant degree (58).

In the study of colon cancer tumor stem cells, Li et al. found that CD133 gene knockout of tumor stem cells not only reduced the proliferation ability of stem cells but also caused the loss of vimentin of cells. Although the cells could not completely lose tumorigenicity, significant inhibition was produced on their tumor characteristics, which could provide a new target for the treatment of colon cancer (59).

### 3.7 A CRISPR gene knockout tool for screening - inflammatory associated bowel disease

About 20% of colorectal cancer cases are closely associated with colitis. Chronic infection and inflammation contribute to the occurrence and development of tumors. Chronic infection factors such as intestinal flora can promote CRC progression through many processes, including chronic inflammatory state and immune response imbalance, interaction with host epigenetic mechanism, change stem cell dynamics and influence host metabolism (60).

The CRISPR method has also been used to screen for IBD-related diseases, mainly through gene knockout, but other commonly used methods include gene editing and gene library screening (**Table 2**).

**Table 2:** CRISPR/Cas9 techniques in new hot spots colon Cancer.

Type	Gene modified	Disease model	References
metabolism genes		colon cancer	
knockout	LDHA and LDHB; NAT1; SDHB; VDR; PPIP5KS; NOXO1; PLAUR; PPIP5K; NHLRC2		(26–32, 139, 140)
immune therapy		colon cancer	
knockout	ILK; AH1; SIAE; CD133		(66, 68, 141, 142)
gene editing	CXCR2 and IL-2		(84)
Inflammatory bowel disease		colon cancer	
knockout	Wnt-1; NHE8; P-cadherin; IL-6; SPHK1; IDO-1		(60–64, 67)
gene editing	P53		(81)
CRISP library	LGALS2; NFKBIZ, ZC3H12A and PIGR;/; TRAF5		(110–113)
Epithelial-mesenchymal transformation		colon cancer	
knockout	Nrp2; Tks4; ALDOB		(69, 70, 143)
knockin	p53 R273H		(71)
SNPs linkage imbalance		colon cancer	
knockout	SNPs Colon cancer risk genes;		(72)
gene editing	rs4779584 and rs1406389; rs34149860; rs6854845		(99–101)

**Figure 4:**
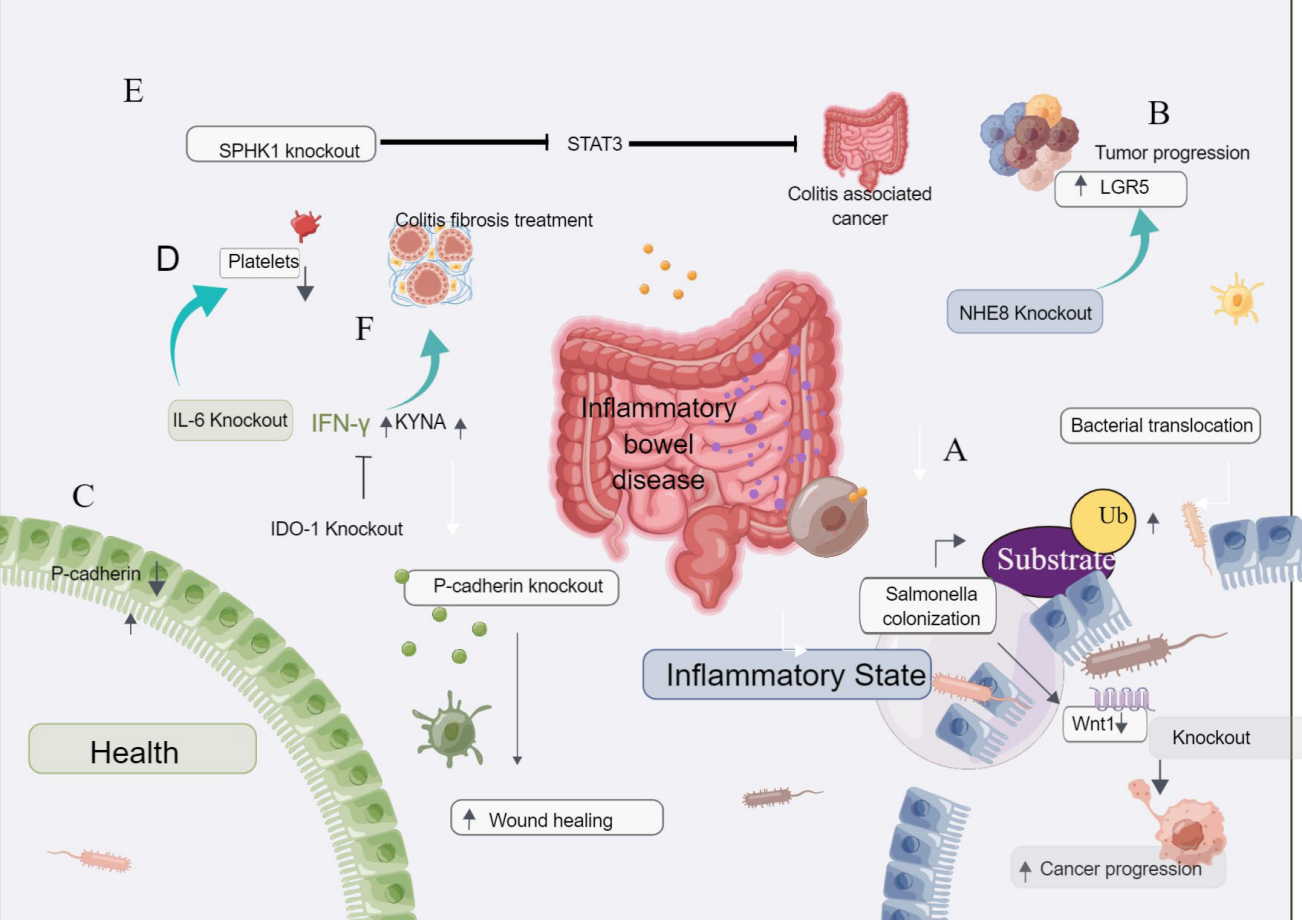
The CRISPR gene knockout tool is used to screen for inflammatory bowel disease. **(A)** Salmonella-induced ubiquitination leads to decreased Wnt-1 levels in tumor cells. CRISPR/Cas9 down-regulation of Wnt-1 affects cancer cell invasion and migration. **(B)** CRISPR/Cas9 knockdown of NHE8 in colon cancer cells resulted in the enlargement of transplanted tumors and increased expression of LGR5, suggesting that NHE8 could inhibit the proliferation of cancer cells. **(C)** P-cadherin is highly expressed in inflammatory bowel disease and inflammatory colon cancer but is lowly expressed in normal mucosa. After the knockout of P-cadherin, injury recovery and wound healing were accelerated. **(D)** IL-6 plays a role in inflammatory colon cancer and plays a key role in the process of platelet increase. After the knockout of IL-6, the number of platelets decreased, suggesting that IL-6 is related to the increase of platelets in inflammatory colon cancer. **(E)** Loss of intestinal epithelial SPHK1 prevents the development of colitis-related cancers by inhibiting epithelial STAT3 activation. **(F)** Knockdown of IDO-1 can affect the therapeutic effect of interferon-γ and KYNA, suggesting that IDO-1 is a good therapeutic target for colitis and fibrosis.

Wang et al. found that salmonella may increase the risk of cancer, with decreased expression of Wnt-1 in malignant tumors. Wnt-1 signal transduction is closely related to inflammation and tumorigenesis. Both *in vitro* and *in vivo* studies have shown that salmonella can reduce Wnt-1 levels in intestinal epithelial cells, and the downregulation of Wnt-1 levels in cancer cells is associated with cancer progression. Thus, salmonella-induced ubiquitination leads to decreased Wnt-1 levels in tumor cells. CRISPR/Cas9 down-regulates Wnt-1, thereby affecting the invasion and migration of cancer cells **Figure 4A** (60).

Xu et al. found that NHE8 could inhibit the expression of LGR5 in the colon and HT29-derived tumors and colon tissues. NHE8 has a protective effect on the intestinal mucosa. When NHE8 was knocked out in colon cancer cells by the CRISPR/Cas9 system and injected into mice, the tumors formed in mice were larger than those formed by wild-type tumor cells injected into mice, suggesting that NHE8 can inhibit the proliferation of cancer cells. After the NHE8 gene knockout, the expression of LGR5 was increased, suggesting that LGR5 was correlated with the expression of NHE8, and NHE8 regulated the expression activity of LGR5 **Figure 4B** (61).

Naydenov et al. showed that P-cadherin was highly expressed in inflammatory bowel disease and inflammatory colon cancer, while it was low expressed in normal mucosa. After P-cadherin knockout, injury recovery and wound healing were accelerated **Figure 4C** (62).

Josa et al. described the mechanism of thrombocytopenia in inflammatory colon cancer. IL-6 plays a role in inflammatory colon cancer and plays a key role in the process of platelet increase. After the knockout of IL-6, the number of platelets decreased, suggesting that IL-6 is related to the increase of platelets in inflammatory colon cancer **Figure 4D** (67).

Park et al. found that the deletion of sphingosine kinase 1(SPHK1) could inhibit the activation of STAT3, thus inhibiting tumor proliferation. Loss of intestinal epithelial Sphk1 prevents the development of colitis-related cancers by inhibiting epithelial STAT3 activation **Figure 4E** (63).

Human adipose-derived mesenchymal stem cells (HADSCs) can treat colitis fibrosis, but their efficacy is inhibited due to their low reactivity and inhibition. The expression and secretion of human interferon-γ and KYNA combined livestock treatment HADSCs promote indoleamine 2,3-dioxygenase-1 (IDO-1) signaling, effectively improving the fibrosis of colitis and colon. After knocking out IDO-1, its therapeutic effect decreased, suggesting that IDO-1 can increase the treatment effect of interferon-γ and KYNA, and play a good therapeutic target in colitis and fibrosis **Figure 4F** (64).

### 3.8 A CRISPR gene knockout tool for screening immunotherapy-related genes: Application of CRISPR/Cas9 knockout technique in a new emerging hotspot of colon oncology immune therapy

Tumorigenesis is a multi-step process involving complex interactions between cancer cells and the host immune system (65). In addition to rapid advances in basic oncology research, CRISPR/Cas-mediated genome editing also holds great promise in cancer immunotherapy (**Table 2**).

**Figure 5:**
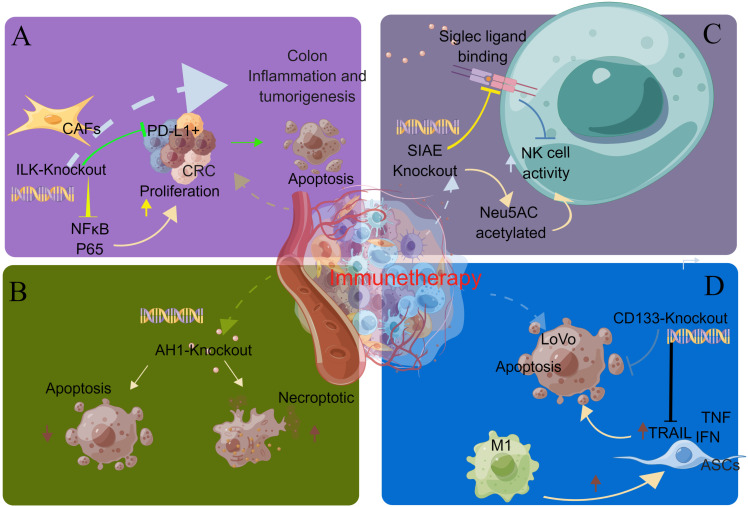
CRISPR gene knockout tool for screening immunotherapy-related genes. **(A)** By knocking out the ILK gene, cancer cells become more sensitive to immune cells. The expression of ILK is correlated with the expression level of PD-L1, which can be used as a therapeutic target for PD-L1-related immune escape. **(B)** In the prophylactic tumor inoculation model, the number of immunogenic necrotic cells was higher than that of apoptotic cells after AH1 knockdown. Necrosis may be the preferred form of ICD in cancer therapy. **(C)** SIAE is a gene that regulates the acetylation of Neu5AC. SIAE gene knockout can improve the acetylation level of Neu5AC and enhance the killing ability of NK cells. **(D)** TRAIL can kill CD133-positive tumor stem cells and reduce the number of M2 macrophages. The expression level of CD133 decreased after treatment. TRAIL can be used as a therapeutic drug for CD133 (+) tumor stem cells.

Integrin-linked kinase (ILK) promotes colon inflammation and tumorigenesis and can be used as a biomarker for colon cancer prognosis and immune cell invasion. Almasabi et al. revealed the relationship between immunosuppression of ILK and TME and the prognosis of colon cancer. Without the ILK gene, cancer cells become more sensitive to immune cells. The expression of ILK is correlated with the expression level of PD-L1, which can be used as a therapeutic target for PD-L1-related immune escape **Figure 5A** (66).

Aaes et al. investigated the mechanism by which expression of immunogenic cell death (ICD) induces AGS-specific antitumor immune responses. The expression of endogenous AH1 tumor AG can mask the intensity of immunogenicity induced by different cell death pathways. In the preventive tumor inoculation model, the number of necrotic immunogenicity cells was higher than apoptosis after AH1 knockout. Necrosis may be the preferred form of ICD for cancer treatment **Figure 5B** (141).

Grabenstein et al. studied the mechanism of the effect of SIAE expression level on the killing ability of NK cells. SIAE is a gene that regulates the acetylation of Neu5AC. SIAE gene knockout can increase the acetylation level of Neu5AC and enhance the killing ability of NK cells **Figure 5C** (142).

M1 macrophages can induce adipose tissue-derived stem cells (ASCs) to overexpress TRAIL, which is an apoptosis-inducing ligand associated with type I interferon and tumor necrosis factor (TNF). TRAIL can kill CD133-positive tumor stem cells and reduce the number of M2 macrophages. The expression level of CD133 decreased after treatment. However, tumor stem cells with loss of CD133 expression are resistant to TRAIL. Eom et al. used gene knockout technology to knock down the CD133 gene of tumor stem cells, and the tumor growth was not inhibited, indicating that TRAIL can be used as a therapeutic drug for CD133 (+) tumor stem cells **Figure 5D** (68).

### 3.9 DNA based knockout/in epithelial-mesenchymal transformation gene

Poghosyan et al. investigated the role of Neuropilin-2 (Nrp2) in tumor proliferation and maintenance of tumor aggressiveness. Neuropilin-2 regulates lymphatic metastasis and production, Using the CRISPR/Cas9 technique to knock out the Nrp2 gene of colorectal cancer cells with mesenchymal phenotype, the mesenchymal transformation into epithelium was found with *de novo* dependence on insulin receptor (IR) signaling and autophagy. Combined inhibition of IR signaling and autophagy leads to apoptosis of Nrp2-deficient tumor cells, which can provide ideas for the development of new aggressive CRC combination therapy (69).

Szeder et al. demonstrated the mechanism of action of the Tks4 scaffold protein in the development of colon cancer. Knockout of Tks4 protein using the CRISPR/Cas9 system revealed increased motility, decreased adhesion, and increased fibronectin expression of cancer cells, all of which are manifestations of epithelial-mesenchymal transformation. Loss of the Tks4 protein can regulate the epithelial-mesenchymal transition of tumor cells (143).

Hosain et al. used the CRISPR/Cas9 system to introduce tumor cells heterozygous for the p53 R273H mutant into mice and found that epithelial-mesenchymal transition was increased, and the number of tumor stem cells was increased, thereby promoting tumor formation. PDMP could inhibit glucosylceramide synthase, which restored the expression of p53, reversed epithelial-mesenchymal transition, and reduced the number of cancer stem cells in R273H mutant cells. Restoring p53 mutations by inhibiting ceramide glycosylation could be a novel therapeutic approach (70).

Li et al. studied the mechanism of the glycolytic pathway on the progression of colon cancer. By screening glycolysis-related genes in colon cancer, high expression of ALDOB (ALDOB) was detected in colon cancer cells. By gene knockout of ALDOB, the epithelial-mesenchymal transformation was inhibited, and the growth and migration of colon cancer cells were blocked. This suggests that ALDOB deficiency can inhibit the growth characteristics of colon cancer cells by inhibiting epithelial-mesenchymal transformation, and ALDOB can be used as a new therapeutic target, which is of great significance for the treatment and prognosis of colon cancer (71).

### 3.10 Gene knockout – SNPs linkage imbalance screening

Yao et al. found that single nucleotide polymorphisms (SNPs) were associated with an increased risk of CRC. Using the enzyme CRISPR, a risk-associated enhancer was deleted and the gene with altered expression was identified (72).

Silencing, deletion, or mutation of tumor suppressor genes activates oncogenes, leading to tumor initiation and progression. The inactivation of tumor suppressor genes is an important feature of cancer occurrence and progression (73). Notably, the application of the CRISPR/Cas9 system has enabled rapid validation of tumor suppressor genes *in vitro* and *in vivo*, transforming a revolution in cancer research.

Cancer cells tend to be prone to the “Warburg effect,” which promotes glycolysis or aerobic glycolysis, even when oxygen is in plentiful supply (74). Glucose metabolism disorders, lipid metabolism abnormalities, amino acid metabolism abnormalities, mitochondrial biogenesis, and other biological energy metabolism abnormalities including metabolic reprogramming pathway is also present in cancer cells (131, 132). Understanding the mechanisms of energy metabolism may provide new ideas for targeting energy generation pathways in cancer therapy.

Cancer stem cells (CSC) play a crucial role in the process of tumor initiation, progression, recurrence, and drug resistance. CSC may be derived from the reprogramming of oncogenes and the dynamic properties of cancer cells; the identification of CSC-related genes is expected to generate new cancer therapeutic targets. Currently, the application of CRISPR technology in tumor stem cells provides a new direction for clinical tumor therapy.

Carcinogenesis includes the acquisition of function mutations of oncogenes and the loss of function mutations of tumor suppressor genes. Most molecularly targeted therapies are oncogene inhibitors; however, in cancer, tumor suppressor genes are altered more frequently than oncogenes. In recent years, several promising strategies have emerged to target tumor suppressor genes or the pathways controlled by these genes (46, 138). Here, we describe the evolution of CRISPR approaches aimed at treating tumors driven by inactivated tumor suppressor genes.

## 4 CRISPR/Cas9 gene editing

### 4.1 Study of tumor stem cells using CRISPR/Cas9 gene editing

**Figure 6:**
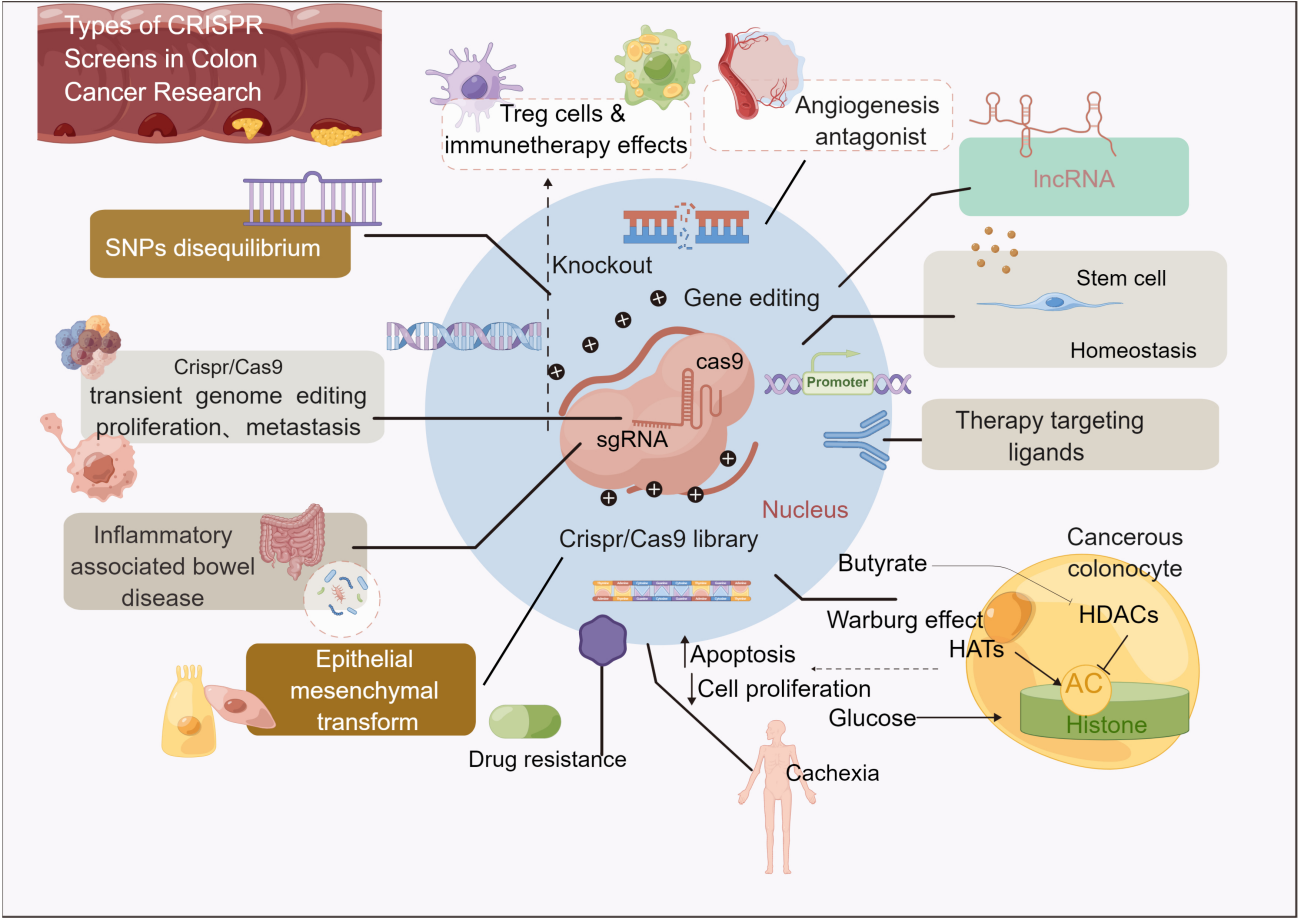
General overview of the article.

With the rapid development of biotechnology based on Cas9 (**Figure 6**), a number of Cas9-based clinical trials may refer to the editing of extracellular somatic cells and the future use of patients. CRISPR/Cas9 gene editing system has been widely used in basic cancer research. By specifically correcting mutations, some progress has been made in inhibiting the growth and progression of colon cancer (75, 76).

The key to targeted cancer therapy is to understand the gene drivers of cancer formation. Han et al. investigated RSPO fusion associated with recurrent colon cancer, using CRISPR/Cas9 genome editing to precisely reconstruct chromosomes in mice, and induce EIF3E-RSPO2, PTPRK-RSPO3 fusion rearrangements, it was found that Rspo2 and Rspo3 fusions are completely Wnt-dependent and can independently initiate tumor development and progression (131).

Ascenção et al. introduced multiple gene mutations into normal colonic epithelial cells to establish the CRISPR/Cas9 genome editing system and investigate whether multiple gene mutations affect H2S production enzymes. Studies have shown that the accumulation of genetic mutations in colon epithelial cells is associated with the progressive upregulation of the H2S production pathway, resulting in more aggressive colon cancer (77).

Ke et al. studied the mechanism of tissue-specific super-enhancer (SE) and inner variants in established GWAS loci. The results showed that the SNP rs11064124 at 12p13.31 was significantly correlated with the risk of colon and rectum adenocarcinoma. Rs11064124-g could increase the activity of enhancers and activate the transcription of tumor suppressor genes CD9 and PLEKHG6, which provided new insights into the pathogenesis of colon cancer (78).

The mutation of the KRAS gene promotes the activation of the MAPK pathway, which promotes the growth of colon cancer cells. Abnormal activation of the PI3K pathway activates the inhibition of the MAPK pathway in CRC with KRAS mutation. Therefore, the Inhibition of MAPK and PI3K pathway is an effective treatment for KRAS-mutated colon cancer. Wang et al. used CRISPR/Cas9 to establish a quadruple deletion system of KRAS, MEK1, PIK3CA, and MTOR genes. Quadruple editing can significantly inhibit the activation of MAPK and PI3K pathways in KRAS mutant CRC and inhibit tumor growth (80).

TP53 gene mutation can improve the malignant potential of colon cancer. Watanabe et al. first demonstrated that TP53 gene mutation improves the invasiveness and proliferation of tumor cells. Two types of mutated TP53 cells were generated *via* the CRISPR/Cas9 system, both of which exhibit blocked apoptosis (81).

The organic model of sporadic early-onset colorectal cancer (EOCRC) is not perfect enough. Yan et al. studied key mutations and transcriptional changes in an EOCRCs-rich organic biobank to supplement the existing model and found that the model contained the PTPRK-RSPO3 fusion gene. The RSPO3 fusion gene, which is transcriptionally similar to the normal gene, evolved into a mature organism, and the CRISPR/Cas9 gene-editing APC mutation kept the cell in the progenitor phase (79).

Matas et al. also applied a single molecule mutation detection method based on CRISPR-DS technology and found that there were more abundant gene mutations in the normal colon of colon cancer patients than that of non-colon cancer patients, including oncogenic KRAS mutations and TP53 mutations, and pathogenic gene mutations were prevalent in normal somatic cells of cancer patients (82).

Shailes et al. established a human cell model of APC mutant CRC by CRISPR/Cas9 gene editing to search for new therapeutic strategies. HMG-CoA reductase (HMGCR) inhibitors can promote apoptosis by inhibiting Wnt signaling. After statin therapy, the expression level of the anti-apoptotic protein survivin was decreased only in APC mutant cells (83).

### 4.2 A CRISPR gene editing tool for screening immunotherapy-related genes

Natural killer cells (NK) play an important role in killing tumor cells, but in colon cancer, these cells are less infiltrated and less active, so their ability to kill tumor cells is limited. NK cells migrate to tumor sites through chemokines secreted by tumors, and up-regulating chemokine receptors of tumor cells is a key step to improving the killing ability of NK cells. Gao et al. overexpressed NK-92 chemokine receptor CXC, chemokine receptor 2 (CXCR2), and cytokine interleukin (IL-2) through CRISPR/Cas9 gene editing technology, indicating that NK cells had enhanced killing ability and inhibited tumor growth (84).

### 4.3 Applications of CRISPR gene editing tools for diagnosis and therapy of colon cancer

Choi et al. found that RNA-guided CRISPR/Cas12A endonuclease has the potential to be a therapeutic tool for colon cancer. Cas12A has a recognition preference for T-rich PAM, which is modified and fused by introducing mutations to form engineered LBCAS12A and LBABE8E variants. A single target base can be directly targeted, but its role in therapeutic editing is limited. Due to the narrow targeting window and the limitation of PAM recognition specificity, the LbABE8E base editor can expand its targeting range by altering PAM specificity, making it a potential genome-editing tool for targeted sites for cancer editing therapy (85).

Selective deletion of mismatch repair gene MLH1 in human colonic organoids using CRISPR/Cas9 molecular scissors resulted in the accumulation of mutations that precisely matched the mutation burden in human mismatch repair deficient colorectal cancer (86).

Li et al. used CRISPR/Cas9 and the single-guide RNA (sgRNA) system to construct gene editing tools to edit mutations in beta-catenin driver genes and restore their normal biological functions. The β-catenin gene mutation has carcinogenic activity, and the growth of tumor cells is slowed down after mutation correction, which provides a new method for cancer gene therapy (87).

Zhang et al. constructed HSV oncolytic virus by CRISPR/Cas9 technology, genetically modified HSV-1 genome, and the combined application of IL12 and CXCL11 in the oncolytic virus provided a new idea for the treatment of colon cancer (88).

The abnormal expression of MUC5AC is secretory mucin, which plays a certain role in the development of colon cancer and the generation of drug resistance. Moreover, the up-regulation of MUC5AC expression can affect the expression of the CD44/β-catenin/p53/p21 gene, thus reducing the sensitivity to 5-FU, oxaliplatin, and other chemotherapy drugs. Pothuraju et al. carried out MUC5AC gene knockout in CRC cells through RNA interference and CRISPR/Cas9 mediated system, conducted *in vitro* functional determination, and constructed a mouse gene knockout model *in vivo*, The mechanism of MUC5AC in tumorigenesis was studied and the cause of drug resistance was elucidated (89).

Chakroborty D et al. found that targeted NPY/Y2R can treat colon cancer and independently regulate angiogenesis within CA. After the VEGF-A gene was knocked out using CRISPR/Cas9 gene editing technology, angiogenesis was inhibited in mice treated with a Y2R antagonist (90).

RNA binding protein HuR (ELAVL1) can enhance the expression of HuR, and apoptosis is increased after the knockout of HuR using CRISPR/Cas9 technology. Therefore, HuR can be used as a therapeutic target for colon cancer (91).

Takei et al. studied the mechanism of ERO1α promoting the growth of colorectal cancer and found that ERO1α was highly expressed in colorectal cancer with poor prognosis. After knocking out ERO1α, it was found that the proliferation of colon cancer was blocked, and the cell movement and migration ability were reduced by weakening the expression of integrin-β1 on the cell surface (92).

Ngamkham et al. found that overexpression of pyruvate carboxylase (PC) can promote the progression and metastasis of colon cancer, leading to poor prognosis and shorter survival. After the CRISPR technology was used to knock out PC, tumor growth was inhibited (93).

Oh et al. found that tubulin acetyltransferase αTAT1 regulates the expression of Wnt1, thereby inducing microtubule acetylation and enhancing the proliferation and invasion of malignant tumors. Knockout of αTAT1 using the CRISPR/Cas9 system reduced tumor invasion and inhibited the progression of colon cancer (94).

Membrane-associated loop CH protein 2(MARCH 2) is involved in autophagy regulation and vesicle transport. Xia et al. found that MARCH 2 was highly expressed in colon cancer with poor prognosis and the survival rate of patients was low. After gene knockout of MARCH 2, endoplasmic reticulum stress was activated, cancer cell growth was inhibited and apoptosis was induced (95).

Gunes et al. used CRISPR to up-regulate Klotho gene in colon cancer cell Caco-2, and found that cell proliferation was inhibited and tumorigenic recovery was achieved, confirming that Klotho gene can promote tumor cell apoptosis (96).

Chen et al. found that FAPP2 can regulate the Wnt/β-catenin signaling pathway and promote the growth of tumor cells. FAPP2 is highly expressed in colon cancer cells and promotes the high expression of colon cancer cells. After knocking out FAPP2 by CRISPR/Cas9 technology, tumor cell growth was inhibited and tumorigenicity was reduced (97).

Li et al. studied the mechanism of CLCA1 in the development of colon cancer. After knocking out CLCA1 using CRISPR technology, the proliferation and metastasis of colon cancer tumor cells were enhanced, and the high expression of CLCA1 led to the suppression of Wnt signal transduction and EMT process, thus inhibiting the growth of tumors (98).

### 4.4 The CRISPR/Cas9 gene editing technique was used to study SNPs linkage disequilibrium

Ortini BK studied risk-related SNPs with an imbalanced link to the SNP rs4779584. The loss of this enhancer or another enhancer previously reported in this region was associated with decreased GREM1 expression after CRISPR/Cas9 genome editing, and the 15q13.3 region contained at least two functional variants. They locate different enhancers and influence CRC risk by modulating GREM1 expression. The correlation between rs1406389 and the GREM1 expression quantitative trait locus in the transverse colon further confirmed that GREM1 is one of the targets of these enhancers (99).

Downregulating of cohesive protein SA-1 is associated with the occurrence of colon cancer, especially in African Americans. Datta et al. conducted gene editing of candidate SNP using gene editing technology and observed that rs34149860 SNP was significantly correlated with the expression of SA-1 in colic mucosa, and showed significant racial differences. It was also proved that the presence of miR-29b inhibitors had a great impact on the expression of SA-1 (100).

Cong et al. found that SNP rs6854845 may be a risk factor for colon cancer. By using CRISPR/Cas9 to construct G>T mutant rs6854845 in FHC, HCT-116, and SW-480 cells, and by observing the difference in the tissue pattern of rs6854845 gene between normal colon epithelial and colon cancer, it was found that SNP rs6854845 located in SE (chr4:75.7M-76.0M) could affect the transcription of these genes by disrupting the long-range chromosomal interaction between SE and the target gene cluster (101).

### 4.5 A CRISPR gene editing technique for screening - inflammatory associated bowel disease

Moreover, The CRISPR/Cas9 gene editing tool was also used to model P53-related inflammatory phenotypes. Watanabe et al. found that although scattered tumors in ulcerative colitis were affected by inflammation, their phenotypic changes and inflammatory signal changes would return to the original state after the removal of inflammatory factors, but their essence would not change. This suggests that tumor cells are highly plastic. They used the CRISPR/Cas9 system to model inflammation-associated TP53 mutations, suggesting that chronic inflammation can inhibit the proliferation and stabilization of these cancer cells through the action of p53 (145).

Oncogenes, tumor suppressor genes, chemical resistance genes, metabolic genes, and cancer stem cell genes are related to the occurrence and development of cancer. The CRISPR/Cas9 gene editing system is already widely used in basic cancer research and has made certain progress in inhibiting tumor growth and progression by specifically correcting mutations and restoring abnormal gene expression (75, 76).

The Cas9-based biotechnology field is evolving at a rapid pace, with multiple Cas9-based clinical trials underway or about to begin, and the results may guide the future use of somatic cell editing *in vitro* and patients.

## 5 Screening of functional genes in colon cancer cells using CRISPR/Cas9 library

Identifying the genes that drive the evolution of tumors can clarify the initiation and progression of cancer (146). Mass genomic screening is a powerful tool for detecting mutated genes that reveal phenotypic changes following drug therapy or other stimuli, leading to the identification of new targets for cancer therapy (**Table 1**).

### 5.1 Screening of drug resistance and targeted genes of colon cancer using CRISPR/Cas9 genomic screening

Gao S et al. revealed 44 genes necessary for colonic CSC-enriched spheroid reproduction. The inclusion of key genes of cholesterol biosynthesis (HMGCR, FDPS, and GGPS1) suggests that cholesterol synthesis can be used as a therapeutic target for colon cancer, and will have a better therapeutic effect when combined with conventional chemotherapy (102).

MEK inhibitors, drugs that target the KRAS pathway, are tolerated by colon cancer, YU et al. demonstrated why colon cancer develops resistance to MEK inhibitors. A CRC cell model carrying KRAS mutation was made and CRISPR was used for genome-wide knockout screening to search for drug-resistant genes of colorectal cancer with MEK inhibitors. Finally, it was determined that GRB7 made colon cancer resistant to MEK inhibitors through the PTK pathway. The GRB7-PLK1 axis acts to reactivate the MAPK pathway and is thought to be the main cause of durability. GRB7 is an interacting kinase of PLK1. Therefore, the combination of PLK1 inhibitor and MEK inhibitor is promising to remove the resistance of MEK inhibitor, which will provide a new therapeutic target for KRAS-mutated colon cancer (103).

Zhou et al. found that the expression pattern of histone modification factors is related to drug resistance of tumor cells, and there is tumor heterogeneity among patients. Through genome-wide CRISPR library screening, they found that the ZEB2 gene is related to drug resistance of 5-FU. Understanding the characteristics of the expression patterns of histone-modified proteins is helpful to better understand the mechanism of colonic drug resistance and provide a way for individualized treatment (104).

Additionally, β-catenin is closely related to the proliferation, differentiation, metastasis, and angiogenesis of colon cancer. Zhao et al. found β-catenin-related target genes through gene library screening and proved that these related genes play a role in colon cancer, and can be used as a target for colon cancer treatment to predict the prognosis of colon cancer (105).

In addition, KRAS oncogene mutations are relatively common in the formation of colon cancer, and direct targeted therapy of the KRAS gene is ineffective. Martin et al. found that the KRAS gene had mitochondrial passage-dependent properties, and through whole gene library screening, they found that the growth of cancer cells with KRAS gene mutations required mitochondrial proteins. Therefore, inhibition of the mitochondrial pathway can inhibit the growth of KRAS gene mutant cancer cells, which provides a new therapeutic target for the treatment of K-ras mutant colon cancer (106).

Hu et al. used CRISPR/Cas9 gene knockout to screen out nine potential genes related to colon cancer, which could be used as therapeutic targets. The high expression of CCT6A, RHOQ, RRP12, UTP18, DDOST, YRDC, ACTG1, RFT1, and NLE1 could promote the growth of COAD cells. CCT6A, RHOQ, and RRP12 can lead to a low survival rate in patients, while those with high expression of UTP18, DDOST, YRDC, ACTG1, RFT1, and NLE1 have higher survival rates (107).

### 5.2 Study of tumor stem cells using CRISPR/Cas9 library

Genetic interactions of chromatin regulatory factors (CRS) are associated with drug resistance in cancer. Chen et al. used CRISPR/Cas9 to screen the genetic interactions of CRS associated with drug response in cancer, and established a CRS gene interaction map, providing a method for guiding rational drug use (108).

Šuštić et al. performed a genome-wide screen in KRAS mutant colon cancer cells and found ERN1 to be sensitive to MEK inhibition. To understand how ERN1 regulates the response of tumor cells to MEK inhibitors, they used CRISPR technology to generate ERN1 knockout KRAS mutant colon cancer cells and performed a genome-wide screen. To identify the genes that confer resistance to MEK inhibition, they found that the ERN1-JNK-Jun pathway is a novel regulator of KRAS-mutated colon cancer response to MEK inhibitors, providing a new treatment option for colon cancer resistance to MEK inhibitors (109).

### 5.3 Genome-wide CRISPR tools for screening - inflammatory-associated bowel disease

Reactive oxygen species (ROS) play an important role in tissue inflammation and tumorigenesis. Li et al. used genome-wide CRISPR gene knockout screening to systematically identify genes involved in oxidative stress regulation, it was found that more than 600 GRNAs were highly enriched in surviving cells after a sublethal H2O2 attack. These include GRNAs LGALS2-encoding glycan-binding protein galectin2 (gal2) that targets LGALS2, this protein is mainly expressed in the gastrointestinal tract and down-regulated in human colon tumors. GAL2 inhibits the development of colon cancer and has therapeutic potential in colon cancer (110).

Nanki et al. identified a unique pattern of somatic mutations in ulcerative colitis, where the inflammatory epithelium accumulates somatic mutations in several genes associated with IL-17 signaling, including NFKBIZ, ZC3H12A, and PIGR. However, these genes are rarely affected in colon cancer. These mutations may promote the development of inflammatory processes and have been shown to resist IL-17 signals-induced apoptosis by undifferentiated CRISPR knockout screens (111).

In addition, Guo et al. have provided a comprehensive approach based on genetic and environmental networks to systematically discover relevant modules for the causal determinants of inflammation-induced tumorigenesis. Genome-wide gene prediction methods were used to integrate clinical and web-based results to prioritize candidate genes and to identify functional collaboration modules using CRISPR/Cas9 co-screening. The discovery of differential genetic interactions in which inflammation co-promotes and co-inhibits tumorigenesis have deepened the understanding of the mechanisms by which inflammation promotes tumorigenesis (112).

The total RNA content of N6-methyladenosine (M6A) and its key methyltransferase METTL3 expression in colon cancer tissues of oxaliplatin (OX) resistant patients were higher than those of OX-sensitive patients. Lan et al. performed genome-wide CRISPR screening and revealed that TRAF5 was involved in METTL3-induced resistance. In addition, M2-polarized TAMS was found to enhance OX resistance in cells by enhancing MettL3-mediated M6A modification. Thus, M2-TAMs are the key to the development of resistance (113).

## 6 Using novel Crispr/Cas 9 technology to explore other areas of research in colon cancer

### 6.1 CRISPR/Cas9 and SiRNA-mediated gene ablation to study the immune mechanism of colon cancer

Harryvan et al. investigated the mechanism by which cancer-associated fibroblasts (CAFs) reduce patient survival and are involved in regulating the killing ability of CD8+T cells. Using CRISPR/Cas9 and SiRNA-mediated gene ablation techniques, they studied the processing of the synthetic growth peptide SLP to determine the mechanism involved in the cross-presentation of antigens mediated by fibroblasts. The cross-presentation of neoantigens by CAFs was stronger than that of normal colon fibroblasts. It was found that CAFs can reduce the cell-killing ability and activation ability of CD8+T cells because CD8+T cells have homologous inhibitory interaction with CAFs (147).

### 6.2 Application of CRISPR/Cas13 for RNA targeting in colon cancer

Long non-coding RNA colon Cancer-associated Transcript 1(CCAT1) is very sensitive to BET inhibition. McClellan et al. screened key factors regulating colon tumor growth through a gene library and found that BRD4 (bromodomain and extra terminal BET) protein BRD4 played a regulatory role in colon cancer proliferation, BET inhibitors could be used as tumor growth inhibitors, and CCAT1 could be used as biomarkers of BET inhibitors (148).

Salinas et al. studied the mechanism of long-chain non-coding RNAs (lncRNAs) in cancer and found that they were associated with poor prognosis of cancer. SNHG15 (a bifunctional MYC-regulated noncoding locus encoding a lncRNA) is potentially carcinogenic. SNHG15 is highly expressed in colon cancer cells, especially in cancer cells with high MYC expression. When SNHG15 is knocked out by siRNA or CRISPR/Cas9, the tumor-causing ability and proliferation and invasion ability of cancer cells were reduced, and the sensitivity to 5-FU was enhanced. Overexpression of SNHG15 can regulate AIF activity, thereby promoting tumor progression and increasing drug resistance. SNHG15 can be used as a prognostic indicator and therapeutic target for colon cancer (149).

### 6.3 Pathological model was constructed based on CRISPR/Cas9 genome editing technology

Existing mouse models of colorectal cancer are subject to multiple limitations. Ectopic tumors do not fully reflect the natural substrate environment of the intestinal mucosa. Roper et al. delivered viral vectors carrying CRE recombinases, CRISPR/Cas9 components, CRISPR-engineered mouse tumor organs, or human tumor organs to mice. Through the guidance of colonoscopy mucosal injection-induced mice, the distal colon tumor mouse model can simulate the development process, tumor, tumor stroma interaction mechanism, etc (150).

Lanagan established a mouse model of serrate CRC using CRISPR/Cas9 genome engineering to sequentially introduce genetic alterations associated with serrate colon cancer, which helped to understand the mechanism of serrate colon cancer (151).

### 6.4 Study of tumor stem cells using CRISPR-CHAP-MS (mass spectrometry-based proteomic capture of proteins)

Huang et al. used CRISPR-CHAP-MS (Mass spectrometry-based proteomic capture of proteins) to study the binding protein of the MACC1 promoter region of colon cancer. MACC1 expression induced metastasis of colon cancer, and the binding of HNF4G and PAX6 to the promoter was verified. The proteins associated with MACC1 promoter binding were different in colon cancer cells with different metastatic potentials (152).

## 7 Discussion

Colorectal cancer is the fourth leading cause of cancer death worldwide. The development of new high-throughput methods over the past decade or so, including CRISPR genome-wide and chemical approaches, has allowed the identification of actionable targets of resistance and the development of relatively specific inhibitors for both classical chemotherapeutic agents and new targeted agents. To be used to resensitize resistant tumors (in combination with chemotherapy) or to be synthetically lethal to tumors with specific oncogenic mutations.

Few technologies have had a significant impact in a short period since their discovery, especially in the area of translational health (153). Indeed, CRISPR/Cas9 has fundamentally affected many fields, such as agriculture, biotechnology, and biomedicine, but none has had a more profound impact than cancer research. What’s more, the CRISPR/edit cas9 gene technology has a broad prospect in cancer therapy (**Figure 6**). However, in the technology that can be used safely and effectively before the clinical treatment of cancer, there are still some challenges.

For the safe use of CRISPR/Cas9, improved tools for specificity and off-target detection are needed. Researchers have made many efforts to improve its specificity, including by integrating multiple factors to develop more priority sgRNA designers. Currently, techniques have been developed to detect low-frequency mutations, such as GUIDE-Seq (154), CIRCLE-Seq (155), and CHANGE-seq to control CRISPR/Cas9 specificity and improve the sensitivity of off-target detection. Second, there is a clinical need for safe and efficient delivery of CRISPR/Cas9 components for tissue - and cell-type specific gene editing.

In addition, a comprehensive genomic analysis is necessary to identify cells with normal genomes before clinical application. Because target mutagenesis occurs frequently in single-guided RNA/Cas9-induced double-strand breaks, remote transcriptional consequences will be induced and may have pathogenic consequences (156). Therefore, CRISPR/Cas9 activation technologies requiring precise Spatio-temporal control in cellular and complex conditions such as cell-specific promoters, small molecule activation/inhibition, biological response delivery vectors, and optical/ultrasonic/thermal/magnetic activation of the CRISPR/Cas9 system (157).

Finally, there is a humoral and cell-mediated adaptive immune response to Cas9 in humans, which may compromise the efficiency of gene editing. Therefore, optimization of the carrier, dose, dosing way and immunosuppression is the perfect CRISPR/Cas9 gene editing potential method in the body (158).

For now, the biggest obstacle to using CRISPR/Cas9 in the body is the delivery system. The CRISPR/Cas9 cargo and delivery vehicles reported so far include physical delivery methods (e.g., microinjection; Electroporation), viral delivery methods (e.g., adeno-associated viruses; Full-size adenoviruses and lentiviruses) and non-viral delivery methods (e.g., liposomes; Complex; Gold particles), all have their relative advantages (159).

Viral vectors are the most common CRISPR/Cas9 delivery vectors. Non-viral vector is a new research field and has many advantages compared with viral vector. Non-viral vector systems include lipid nanoparticles, cell-penetrating peptides (CPPS), DNA “nanowires”, and gold nanoparticles. In addition, a number of delivery technologies are still in the experimental stage, including streptococcolysin O, multifunctional coated nanodevices (MEND), lipid-coated mesoporous silica particles, and other inorganic nanoparticles.

CRISPR/Cas technology is an era of unprecedented development in cancer research, diagnosis, and treatment, and some efforts are still needed to overcome these technical bottlenecks.

CRISPR/Cas9 technology has transformed the study of genetic pathways that control cell differentiation and function. Recent advances have enabled the application of these methods to immune cells and accelerated the pace of immunological functional genomics. This review summarizes recent advances in CRISPR/Cas9 technologies in the field of colon cancer immunity and shows how they can be used to discover novel genetic regulators that manipulate the immune system. Including the recently reported role of the ZG16 protein in tumor immune regulation, CRISPR may elucidate its regulatory mechanism in tumor immunity in the future (160). Immunological research over the next decade will heavily leverage early applications of this technology to continue to reveal new mechanisms and pathways that span different immune cell types and functionally related biological systems. Furthermore, the combination of CRISPR screens with single-cell transcriptome and epigenetic read-offs could expand the utility of these methods (161, 162).

Spontaneous deamination of cytosine is the main source of C•G to T•a transformation account for half of all known pathogenic point mutations in humans. Thus, the ability to efficiently convert the target A•T base pair into G•C could facilitate the research and treatment of genetic diseases. Base editing allows irreversible direct conversion of base pairs in the target genome and can be performed more efficiently and with fewer unwanted products (such as random insertions or deletions (indels) or translocations) than standard genome editing methods that introduce point mutations. Adenine base editing (ABEs) that mediates the A·T to G·C transformation in genomic DNA. When fused with catalytically damaged CRISPR-Cas9, the target A·T is effectively converted to G·C base pairs at a very high product purity (usually 99.9%) and a very low indels rate (usually 0.1%). Compared with Cas9, point mutations are introduced cleanly, induce less untargeted genomic modification, and can be installed in human cells with disease-correcting or disease-suppressing mutations (163).

Of course, there are some very valuable studies on colon cancer that involve CRISPR technology, such as the application of CRISPR in Lynch syndrome (LS). Using clustered regular interval short palindromic repeat-Cas9 gene editing to introduce variants into endogenous MSH2 sites in human embryonic stem cells, while eliminating wild-type genes, providing valuable information for determining pathogenic LS variants; using CRISPR/Cas9 system to introduce MSH2 mutations reported in the Lynch syndrome (LS) family into HeLa cells to study the phenotype of MMR deficiency. The function of the hypothetical enhancer was verified by luciferase report analysis, chromatin immunoprecipitation and CRISPR-Cas9-mediated endogenous region deletion (164–166).

CRISPR/Cas9 has evolved into a stable, efficient, simple, and widely used gene-editing technique. Indeed, CRISPR/Cas9 has fundamentally affected cancer research and will continue to have an even more profound impact. The CRISPR/Cas9 technology enables rapid, accurate and detailed study of cancer genomes, opening up new ideas for the study of new mechanisms of tumor development. More importantly, CRISPR/Cas9 gene editing technology offers broad prospects for cancer treatment. CRISPR/Cas9’s specific tool for target detection offers a safe and personalized approach to cancer treatment. Improving the detection sensitivity of CRISPR/Cas9 specificity will help to control the effective, safe and targeted delivery of CRISPR/Cas9 in cells and its application in tumor research, diagnosis and treatment.

CRISPR/Cas9 can identify new drug resistance or sensitivity mutations in colon cancer, develop new therapeutic targets using gene editing technology, and significantly improve the treatment effect of colon cancer patients. This article reviews the rapid development of CRISPR/Cas in colon cancer, including gene editing, transcriptional regulation, gene knockout, genome-wide CRISPR tools, therapeutic targets, stem cell genomics, immunotherapy, metabolism-related genes and inflammatory bowel disease applied in colon cancer. Finally, the limitations and future development of CRISPR/Cas9 in colon cancer research are pointed out. In summary, this article reviews the application of CRISPR/Cas9 gene editing technology in basic research, diagnosis and treatment of colon cancer.

## Author contributions

Conceptualization: HM. Data curation: YL. Methodology: MN, YY, and YD. Project administration: MZ. Resources: HM. Supervision: MZ. Writing – original draft: MN. Writing – review and editing: HM. All authors contributed to the article and approved the submitted version.
